# Linking health facility data from young adults aged 18-24 years to longitudinal demographic data: Experience from The Kilifi Health and Demographic Surveillance System

**DOI:** 10.12688/wellcomeopenres.11302.2

**Published:** 2020-02-27

**Authors:** Christopher Nyundo, Aoife M. Doyle, David Walumbe, Mark Otiende, Michael Kinuthia, David Amadi, Boniface Jibendi, George Mochamah, Norbert Kihuha, Thomas N. Williams, David A. Ross, Evasius Bauni

**Affiliations:** 1 KEMRI/Wellcome Trust Research Programme, Kilifi, Kenya; 2 INDEPTH, Accra, Ghana; 3 London School of Hygiene and Tropical Medicine, London, WC1E 7HT, UK; 4 Imperial College, London, SW7 2AZ, UK

**Keywords:** Record linkage, Demographic Surveillance, Adolescent Health, Kenya

## Abstract

**Background:** In 2014, a pilot study was conducted to test the feasibility of linking clinic attendance data for young adults at two health facilities to the population register of the Kilifi Health and Demographic Surveillance System (KHDSS). This was part of a cross-sectional survey of health problems of young people, and we tested the feasibility of using the KHDSS platform for the monitoring of future interventions.

**Methods:** Two facilities were used for this study. Clinical data from consenting participants aged 18-24 years were matched to KHDSS records. Data matching was achieved using national identity card numbers or otherwise using a matching algorithm based on names, sex, date of birth, location of residence and the names of other homestead members. A study form was administered to all matched patients to capture reasons for their visits and time taken to access the services. Distance to health facility from a participants’ homestead was also computed.

**Results:** 628 participated in the study: 386 (61%) at Matsangoni Health Centre, and 242 (39%) at Pingilikani Dispensary. 610 (97%) records were matched to the KHDSS register. Most records (605; 96%) were matched within these health facilities, while 5 (1%) were matched during homestead follow-up visits.  463 (75.9%) of those matched were women. Antenatal care (25%), family planning (13%), respiratory infections (9%) and malaria (9%) were the main reasons for seeking care. Antenatal clinic visits (n=175) and malaria (n=27) were the commonest reasons among women and men, respectively. Participants took 1-1.5 hours to access the services; 490 (81.0%) participants lived within 5 kilometres of a facility.

**Conclusions:** With a full-time research clerk at each health facility, linking health-facility attendance data to a longitudinal HDSS platform was feasible and could be used to monitor and evaluate the impact of health interventions on health care outcomes among young people.

## Abbreviations

HDSS: Health and Demographic Surveillance System

ID: Identification

INDEPTH: International Network of field sites with continuous Demographic Evaluation of Populations and Their Health in developing countries

## Introduction

Health and Demographic Surveillance Systems (HDSS) provide longitudinal information on populations living within geographically-defined areas, including data on fertility, mortality and migration ^1– 3^. These data can be useful to public health policy makers both locally and internationally ^2– 5^. When HDSS data are combined with data from health services, they can be used to monitor and evaluate outcomes of research and health care programmes ^1– 3^.

Data linkage is a process of pairing records from two data sources or bringing together information from two records that relate to the same individual or entity ^6– 8^. This linkage process frequently involves the use of basic socio-demographic indices that uniquely identify an individual across two or more datasets ^8^. Although there is growing interest in integrating HDSS and health service data ^3^, examples of how this can be performed are relatively rare ^5, 9– 11^.

The Kilifi Health and Demographic Surveillance System (KHDSS) was established in 2000 to monitor births, deaths, pregnancies and migration events within a sub-population of Kilifi County on the coast of Kenya ^1^. The KHDSS covers an area of 891km ^2^ with a resident population of approximately 280,000 in 2016. The area is served by 1 referral hospital, three health centres, 14 dispensaries and numerous private health service providers. KHDSS data include basic details of all homesteads and the names, dates of birth, sex, national identity (ID) card numbers and ethnicity of all homestead members, and are updated with births, deaths and in- and out-migration events three times a year. A homestead comprises of one or more houses or dwelling units with people, also referred as residents, who have one person they refer to as the head. Geographic coordinates for dwelling units and health facilities are routinely collected using global positioning system technology (GPS). The KHDSS population register has been linked to a surveillance system of children admitted to Kilifi County Hospital since 2001, and linkage was expanded to cover the maternity and adult wards admissions in subsequent years ^1^. Finally, data on childhood immunizations administered at 30 government and private not-for-profit health facilities have been linked to the register since 2008 ^12^. However, data linkage for other age groups attending these peripheral health facilities has not been done. In this study, we linked young adults (18–24 year-olds) attending health facilities within the KHDSS area and asked their reasons for visiting. This exercise was part of the International Network of field sites with continuous Demographic Evaluation of Populations and Their Health (INDEPTH) Healthy Transitions to Adulthood Study (IHTAS), which also aimed to describe the nutritional and health problems of young people aged 13–24 years in Kilifi and Dodowa HDSS, both members of INDEPTH.

Linking data relating to adolescents and young adults who attend such facilities was anticipated to provide a useful platform to monitor and evaluate interventions to improve adolescent health ^13– 15^.

## Methods

### Study area

**Figure 1.**
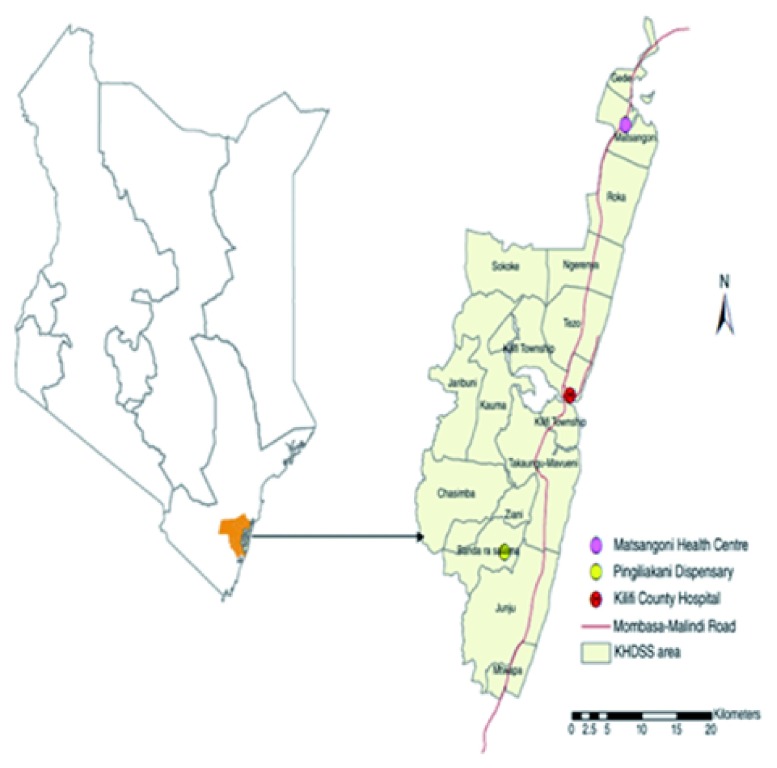
Study area.

The study was conducted within the area served by the KHDSS on the coast of Kenya ( Figure 1) during a 3 month period from mid-August 2014 to end of October, 2014.

A pre-study evaluation was conducted for 6 health facilities within the KHDSS area. The 6 facilities evaluated were spread across the KHDSS area to ensure a fair DSS area representation. The evaluation explored health facility staff willingness to participate in the study, availability of space to run the study, electricity supply and youth friendly services including existence of trained staff on Youth friendly services as well as whether there was a youth corner.

The two facilities that were selected for the survey, Pingilikani dispensary and Matsangoni health centre, met the criteria necessary for the study and their combination was judged to be the most likely to represent the range of facilities. The two facilities are far apart; Pingilikani dispensary is in the southern part with a catchment population fully within the KHDSS while Matsangoni health centre is situated in the northern part of the KHDSS area and its catchment population straddles the boundary of the DSS area. Staff willingness to participate in the study was essential for supporting the study and to fill in the reason for the visits on the study questionnaire. The target participants for the linkage study in the two health facilities were young adults aged 18–24 years who sought health services from the facilities during the study period. We characterised youth-friendly health care services as the availability of health workers who were trained to deal with and handle respectfully and confidentially the heterogeneous health issues of young people ^15, 16^

### Participant recruitment

Data clerks were trained on consenting, matching, and the administration of the study questionnaire ^3^. We used laptop computers that were up-loaded weekly with the most recent version of the KHDSS database to identify and match resident young adults who visited these health facilities. All consenting patients aged 18–24 years were eligible to participate in the study.

All young people aged 18–24 years attending the facilities on the study days were the only eligible for the study and were identified at the reception desks and referred to the study data clerks for assessment of eligibility, consenting, matching, linkage to the population register and collection of study-specific information. The health facility reception desk was the most convenient place for screening since basic demographic details including age are routinely captured there for anyone seeking heath care. At the reception, identification was based on age and not DSS residence. DSS residence was determined during the subsequent matching process. This is because an individual might have not been a DSS resident but was likely to have interacted with the database at other points, either at the county hospital, or at vaccination centres, or might have left the DSS area sometime and now has returned. After obtaining consent, the data clerk searched for the participant’s records in the KHDSS records. Those who were successfully matched were issued with a study form on which the data clerk recorded the facility name, date and time of arrival. The patients then proceeded with these forms to the attending clinicians who recorded the purpose of the visit. Although the main aim of the study was to determine the feasibility of patient linkage at the facilities, we also used the linkage to gain a better understanding of the main reasons why the young people visited the facilities. After completing facility procedures, including the collection of prescribed medications, patients returned their forms to the data clerks who recorded the time of departure. The study also provided an opportunity to assess the average time young people took at the facility seeking care. For those not found on the KHDSS register, details were recorded in a separate form ^17^ for further matching by more experienced experts and to facilitate home visit follow-up. During the home visits, experienced DSS staff identified the homestead with its members, and with the help of other homestead members confirmed whether the participant’s records existed in the KHDSS database.

### Matching

The matching and data-linkage process commenced after written consent had been obtained. We used ID numbers where available, or an algorithm based on national identity numbers, first and second names, date of birth, gender, and ethnicity, and the homestead name, location and sub-location. The KHDSS database algorithm is a search engine that retrieves a set of records that meet the set criteria. This algorithm was applied for this study. The same has also been used for vaccine monitoring in different vaccine centres across the KHDSS area and matching patients’ records with KHDSS data at the Kilifi county hospital. The correct match was obtained by narrowing down using the names of the homestead head, the list of other homestead members, or varying the first 3 letters of names to account for spelling variations. Uncertainties regarding dates of birth were overcome by using a range of options of “+/- 1 year”, “+/-3 years” of the date of birth and “DO NOT KNOW” on instances the person does not know the date of birth

The Euclidian distances to health facilities from the residences of consenting participants were computed using ArcGIS 10.1 desktop software (ESRI, Redlands, CA, USA) from the paired geographic coordinates for the facilities and location of participants’ homesteads. We did not ask participates for the mode or cost of transport or time they travelled to the facility.

## Results

All (n=628) participants who were approached to participate consented, 386 (61.5%) were recruited at Matsangoni Health Centre and 242 (38.5%) at Pingilikani Dispensary. A total of 605/628 participants (96.3%) were successfully matched at the health facilities, a process that took a minimum of 1 minute to a maximum of 3 minutes based on experience of matching patients in the wards and at the vaccine clinics. We failed to match 23 (3.7%) participants at the facility level, who were later followed in the field by more experienced staff and another search was performed with the help of other homestead members. We discovered that some of these participants existed in the database with names different from those they used at the clinic. This process was successful for a further five patients, but remained unsuccessful for 10, while eight patients could not be found in the community.

**Figure 2.**
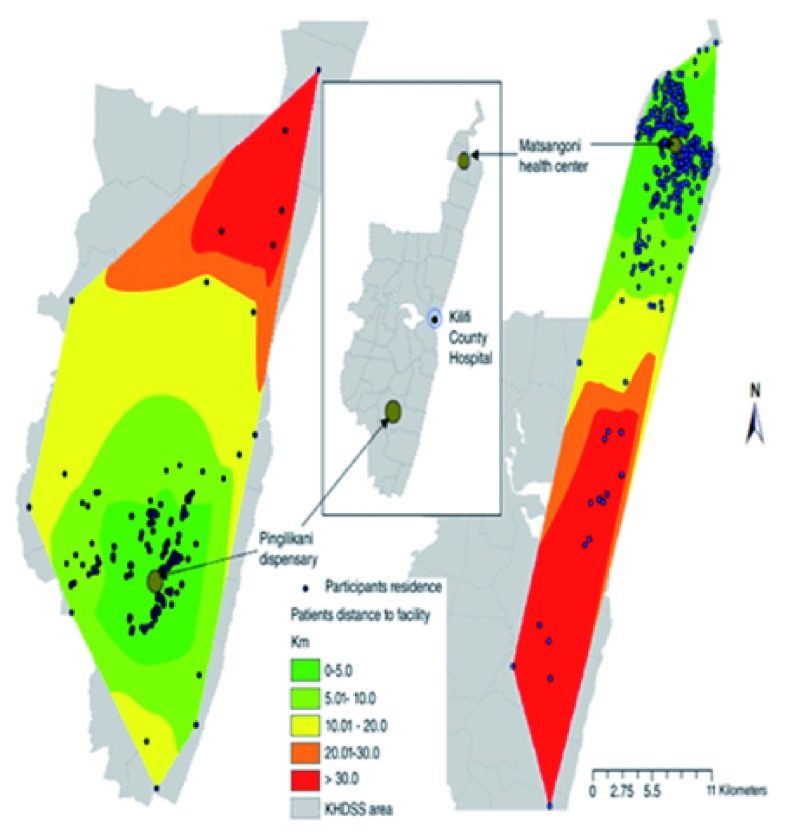
Participants’ distance to health facility from their residence.

Three of the unmatched participants were seen at Pingilikani Dispensary, which is relatively centrally placed, far from the border of the KHDSS area, while the other 15 were seen at Matsangoni Health Centre, which is situated close to the border of the KHDSS area. The homestead visits revealed that none of the 18 participants we failed to match were in the KHDSS population register. Three resided outside the KHDSS area, one was a high school teacher who had recently in-migrated, had given wrong information for the homesteads they said they came from and were not known in these homesteads, another 10 could not be verified because the data collection forms containing details of where they came from were misplaced. However, all who did not match at the facility were excluded from the analysis. The spatial distribution of study participants is shown in Figure 2. 81% of participants lived within 5km or less of the health facilities, and only 3.4% were more than 20 km ( Table 1).

**Table 1.** Distance between participants’ residence and health facilities.

Distance (km)	Matsangoni Health Centre (N=372)	Pingilikani Dispensary (N=233)	Total (N=605	%
0–4	306	184	490	81.0
5–9	43	35	78	13.0
10–19	7	9	16	2.6
≥20	16	5	21	3.4

A total of 715 visits to the health facilities were recorded among the 605 matched participants since some had multiple visits during the study period ( Table 2). Reasons for facility visit differed by gender and there were many more visits by women than men (548/715; 76.6%). The main reasons for visit by women were antenatal clinic (175; 32%) and family planning services (95; 17%) ( Table 2). Malaria (27; 16%) and skin infections (26; 16%) were the commonest reasons for men.

**Table 2.** Overall reasons for health facility visit by gender.

Reasons	Women, N (%)	Men, N (%)	Total, N (%)
Antenatal care clinic	175 (31.9)	1 (0.6)	176 (24.6)
Family planning	95 (17.3)	1 (0.6)	96 (13.4)
Respiratory infection	47 (8.6)	19 (11.4)	66 (9.2)
Malaria	37 (6.8)	27 (16.2)	64 (9.0)
Other non-infectious	39 (7.1)	18 (10.8)	57 (8.0)
Skin infection	21 (3.8)	26 (15.6)	47 (6.6)
Sexually transmitted infection	25 (4.6)	7 (4.2)	32 (4.5)
Gastro-intestinal disease	18 (3.3)	12 (7.2)	30 (4.2)
Urinary tract infection	21 (3.8)	9 (5.4)	30 (4.2)
Ear, Nose and Throat	15 (2.7)	8 (4.8)	23 (3.2)
Injuries	6 (1.1)	15 (9.0)	21 (2.9)
Chest infection	9 (1.6)	7 (4.2)	16 (2.2)
Asthma	6 (1.1)	3 (1.8)	9 (1.3)
Gynaecological disease	8 (1.5)	1 (0.6)	9 (1.3)
Other infections	5 (0.9)	3 (1.8)	8 (1.1)
HIV	11 (2.0)	1 (0.6)	9 (1.3)
Worms	0 (0.0)	6 (3.6)	6 (0.8)
Anaemia	4 (0.7)	1 (0.6)	5 (0.7)
Epilepsy	0 (0.0)	2 (1.2)	2 (0.3)
Abortion	1 (0.2)	0 (0.0)	1 (0.1)
Eclampsia	1 (0.2)	0 (0.0)	1 (0.1)
Mother Child Health	1 (0.2)	0 (0.0)	1 (0.1)
Pregnancy test	1 (0.2)	0 (0.0)	1 (0.1)
Missing	2 (0.4)	0 (0.0)	2 (0.3)
Total	548 (100.0)	167 (100.0)	715 (100.0)

Matsangoni Health Centre had more visits (n=429) than Pingilikani Dispensary (n= 286) ( Table 3). The main reasons for visits differed between the two facilities. The main reasons for visits in Matsangoni Health Centre were antenatal clinic (n=147) or family planning services (n=75), while the main reasons at Pingilikani Dispensary were malaria (n=59), non-infectious conditions (n=36), respiratory infections (n=35), antenatal clinic (n=31) and family planning (n=21).

**Table 3.** Reasons for visits by health facility.

Reasons	Pingilikani, N (%)		Matsangoni, N (%)		Total, N (%)
	Women	Men	Women	Men	
Antenatal care clinic	31 (16.7)	0 (0.0)	147 (40.4)	0 (0.0)	178 (24.9)
Abortion	1 (0.5)	0 (0.0)	0 (0.0)	0 (0.0)	1 (0.1)
Anaemia	2 (1.1)	1 (1.0)	2 (0.5)	0 (0.0)	5 (0.7)
Asthma	1 (0.5)	3 (3.0)	5 (1.4)	0 (0.0)	9 (1.3)
Ear, Nose and Throat	5 (2.7)	5 (5.0)	10 (2.7)	3 (4.6)	23 (3.2)
Epilepsy	0 (0.0)	1 (1.0)	0 (0.0)	1 (1.5)	2 (0.3)
Family Planning	21 (11.3)	0 (0.0)	75 (20.6)	0 (0.0)	96 (13.4)
Gastro-intestinal disease	8 (4.3)	3 (3.0)	10 (2.7)	9 (13.8)	30 (4.2)
HIV	3 (1.6)	0 (0.0)	8 (2.2)	1 (1.5)	12 (1.7)
Injuries	1 (0.5)	11 (11.0)	5 (1.4)	4 (6.2)	21 (2.9)
Mother child health	1 (0.5)	0 (0.0)	0 (0.0)	0 (0.0)	1 (0.1)
Other infections	0 (0.0)	2 (2.0)	6 (1.6)	1 (1.5)	9 (1.3)
Pregnancy test	1 (0.5)	0 (0.0)	0 (0.0)	0 (0.0)	1 (0.1)
Respiratory infection	25 (13.4)	10 (10.0)	22 (6.0)	9 (13.8)	66 (9.2)
Sexually transmitted infection	4 (2.2)	1 (1.0)	21 (5.8)	6 (9.2)	32 (4.5)
Skin infection	8 (4.3)	15 (15.0)	13 (3.6)	11 (16.9)	47 (6.6)
Urinary tract infection	9 (4.8)	7 (7.0)	12 (3.3)	2 (3.1)	30 (4.2)
Chest infection	3 (1.6)	2 (2.0)	6 (1.6)	5 (7.7)	16 (2.2)
Gynecological disease	3 (1.6)	0 (0.0)	5 (1.4)	1 (1.5)	9 (1.3)
Malaria	34 (18.3)	25 (25.0)	3 (0.8)	2 (3.1)	64 (9.0)
Other non-infectious	25 (13.4)	11 (11.0)	14 (3.8)	7 (10.8)	57 (8.0)
Worms	0 (0.0)	3 (3.0)	0 (0.0)	3 (4.6)	6 (0.8)
Total	186 (100.0)	100 (100.0)	364 (100.0)	65 (100.0)	715 (100.0)

The month of September had the highest number of visits (317; 44.3%), followed by October (293; 40.9%). The study was done for a half month in August and the results are not a true reflection for a month’s observation to be compared with the other months. Antenatal care and family planning were the main reasons for health facility visit in October with 80 and 50 visits, respectively, while malaria, non-infectious, respiratory and skin infections were the main reasons in September. Participants took 10 to 480 minutes to be served at the facility, with a median of 80 minutes.

## Discussion

Through this study, we demonstrate that it is possible to link health data collected from the vast majority (97%) of young adults attending two peripheral health facilities to the population register of the KHDSS, using an algorithm with a range of routinely collected personal data. The success rate in matching was similar to linkage conducted in vaccination centres for children under 5 years in the KHDSS area ^12^. A total of 15 of the unmatched patients had sought services in Matsangoni Health Centre, which is located close to the KHDSS border on the Mombasa-Malindi highway. While national ID card numbers facilitated rapid matching and linkage for a few participants, the majority either did not have ID cards with them, or their ID numbers were not on KHDSS database. Although the speed, accuracy and potential of linking individuals using national ID numbers is consistent with similar observations from other studies ^7^, there were particular challenges for this age group. While legally residents are supposed to register for a national ID card on reaching their 18 ^th^ birthday, in practice this is not followed universally. Second, capturing these numbers on the KHDSS database is not immediate as update rounds are only conducted three times a year. Third, not everybody carries their ID cards when seeking health services.

The presence of the participants during the matching process was an advantage because they could quickly identify and confirm other members of their homestead. This speeded up the matching process. On average, in the absence of a national ID card number, it took 2 to 3 minutes to search, match and link an individual. This was reasonably fast and reflects a decade of learning and experience trying to perfect demographic data capture and real-time linkage to clinical data for research in Kilifi, particularly at the Kilifi County Hospital, and also in matching internal migrants. We have also been matching children and mothers in more than 30 facilities that provide vaccinations for the last 8 years with similar matching results ^12^. The search engine used two names only but efficiency, speed and accuracy may improve if more and full names are used in the future ^4, 9, 18, 19^. We used a combination of other variables, which include location of residence, the name of the homestead head and the list of other homestead members for further confirmation. The greater speed and accuracy of matching using ID numbers reinforces the importance of recording these numbers when available, and of encouraging young people to get an ID card after reaching the statutory age of 18 years and carrying it when seeking health services.

Most of the successful matching was done using personal details of names, date of birth and ethnicity in combination with homestead name, name of homestead head and location and sub-location. A sub-location is the smallest administrative unit and several of them constitute a location. Failure to match was partly due to the use of different names from those on the population database, as was confirmed for the 5 who were matched during the home visit.

This study confirmed that it was possible to link individual level health data at peripheral health facilities with the longitudinal population register for young adult residents of KHDSS. This will enhance monitoring of health interventions in the future to inform health policy related to improving the health of young adults within our catchment population. With technological advances, increased accuracy and speed in record linkage may be possible with the adoption of a fingerprint biometric database system, although this is not without its own challenges ^20^.

### Reasons for facility visit

There were substantial differences in the main reasons for clinic visits in young adults by sex, with antenatal care and family planning being the two commonest reasons for women, and malaria and skin infections for men. Differences by sex are likely to be minimal in young children, but diverge after puberty. The substantial differences by health facility partly reflect differences in malaria prevalence across the KHDSS area, with more malaria in the south where Pingilikani dispensary is situated ^21– 23^.

The present findings can be useful to inform and guide adolescent and young adult health programming ^24, 25^. The epidemiological transition is evident in the clinic visit data, where there has been a partial shift away from infectious diseases and undernutrition, with sexually transmitted infections, reproductive health problems and injuries becoming relatively more prominent ^13^. The findings confirm previous work by Bauni and colleagues, who have shown that injuries and pregnancy-related conditions are now major causes of death among young men and women, respectively, living within the KHDSS area ^26^. Although injury and HIV were not among the main reasons for visiting the health facility, other studies of the same community have shown injury and HIV as the main cause of death for young men and women, respectively ^26^. Furthermore, Etyang and colleagues have reported that injuries were the most common cause of admission to Kilifi County Hospital among men, while infectious and parasitic diseases, and pregnancy-related complications were the most common among women ^27^. Similarly, a review of medical causes of admission to hospital among adults in Africa reported injuries to be a leading cause of admission for men, while HIV and pregnancy-related disorders were highest among women ^28^.

The current Kenya health sector strategy recommends that, by 2016, at least 90% of households in Kenya should be within 5km or 1 hour travel time to a public health facility ^29^. This study has shown that 81% of study participants were within 5km or less to the health facility and 6% were more than 10km from the health facility. A high proportion of those with longer distances had temporarily relocated. For instance, some married young women had temporarily moved back to their parents’ homes for care during the postnatal period, while others had temporarily moved for work-related reasons or were students in boarding schools.

It took between half an hour and an hour to receive services. Longer times were usually due to long queues or the need for laboratory tests to confirm the clinical diagnosis. For example, all malaria cases were laboratory-test confirmed.

Strengths of the study included the fact that linking and matching of patient records was done in the presence of the participants who were usually able to confirm that the matching was correct. We also attempted to visit the probable homestead for those who could not be matched at the facility. During the home visits, we found that some people existed in the database with different names, while others did not exist in the KHDSS population register. Matching could be done very quickly (<1 minute) when the participant had their ID card with them and the ID number was in the KHDSS database, but even when this was not the case, the time taken for matching was a maximum of 3 minutes.

However, the study had limitations. Although health facility and KHDSS data were successfully linked for the great majority (97%) of young adults (18–24 years), it was limited to this age group and a small catchment area of two peripheral health facilities over a short period of two and a half months. The two selected facilities are broadly representative of all facilities in KHDSS and it is unlikely that there would have been a significant difference in the success of matching results in other similar facilities. Another study looking at matching in the context of vaccination monitoring found similar high levels of matching ^12^. However, matching would potentially be more challenging in a facility where the staff were less supportive of the study if this prevented study staff from interviewing clients in the reception area. Our focus on the main reason for facility visit precluded the opportunity to determine whether participants had other secondary health problems, an aim that the authors propose to address through future work.

## Conclusions

This is the first study to link outpatient health facility data and KHDSS population data for young adults in peripheral health facilities. The study demonstrated that it was possible to link individual health data on young adults attending the two peripheral health facilities to a longitudinal population register. The main reasons for visiting health facilities identified in this study will inform policy makers on key areas to target for interventions.

These findings show that clinic/KHDSS data linkage is feasible in this context. The combination of the population-based fertility and mortality data from the demographic surveillance system, and the linked health facility data could be the basis for monitoring and evaluation of health care outcomes, demand for health services, and the effectiveness of public health interventions among young adults in this population.

## Ethical approval

Ethical approval for this study was given by the KEMRI/National Ethical Review Committee (Protocol SSC No. 2823: The Health and Health Behaviours of young people (13–24) in Kilifi (version 2.1 dated July 17, 2014)), and by the Research Ethics Committee of the London School of Hygiene & Tropical Medicine (No. 8660). Written consent was obtained from all participants.

## Data availability

### Underlying data

Figshare: Dataset Files 1 and 2 for version 1 of “Linking health facility data from young adults aged 18–24 years to longitudinal demographic data: Experience from The Kilifi Health and Demographic Surveillance System”. https://doi.org/10.6084/m9.figshare.5202850.v1

This project contains the following underlying data:

- Dataset File 1: Linkage data.xlsx
- Dataset File 2: Unmatched participant data.xlsx
  Data are available under the terms of the Creative Commons Attribution 4.0 International license (CC-BY 4.0).

### Extended data

Figshare: Supplementary files 2 and 3 for “Linking health facility data from young adults aged 18–24 years to longitudinal demographic data: Experience from The Kilifi Health and Demographic Surveillance System”. https://doi.org/10.6084/m9.figshare.11880657.v2

This project contains the following extended data:

- SF2: Unmatched participants demographic data form.docx
- SF3: Unmatched Participants.xlsx

Data are available under the terms of the Creative Commons Attribution 4.0 International license (CC-BY 4.0).
