# Supplementary material for: Linking health facility data from young adults aged 18-24 years to longitudinal demographic data: Experience from The Kilifi Health and Demographic Surveillance System
Source: Wellcome Open Res. 2020 Feb 27;2:51. Originally published 2017 Jul 17. [Version 2] doi: 10.12688/wellcomeopenres.11302.2 (PMC7059845; doi:10.12688/wellcomeopenres.11302.2)
Supplement: Peer review history

# Peer review: Reviewer response for version 2

**DOI:** 10.21956/wellcomeopenres.17204.r38047

**Published:** 2020-03-05

**Authors:** Philippe Bocquier

**Affiliations:** 1 Centre de Recherche En Démographie, Université Catholique de Louvain, Louvain‐la‐Neuve, Belgium; 2 School of Public Health, University of the Witwatersrand, Johannesburg, South Africa

These are comments on the revised version of the article. Most of the comments and critiques have been addressed correctly, in particular regarding the methodology (selection of the two health facilities and of the patients). The limitations of the study are now better indicated to the reader. The main remaining bias is related to the non-random selection of the health facilities (and not so much of the patients). The authors are confident that matching results would be similar with other facilities (e.g. with less supportive staff) but this remains to be proven. The results are nevertheless encouraging.

I confirm that I have read this submission and believe that I have an appropriate level of expertise to confirm that it is of an acceptable scientific standard.

# Peer review: Reviewer response for version 1

**DOI:** 10.21956/wellcomeopenres.12195.r26015

**Published:** 2017-10-13

**Authors:** Fiona J Stanley, Hannah C. Moore

**Affiliations:** 1 Telethon Kids Institute, University of Western Australia, Perth, WA, Australia; 2 Wesfarmers Centre of Vaccines and Infectious Diseases, Telethon Kids Institute, University of Western Australia, Crawley, WA, Australia

- Authors should be commended for this work which demonstrates the feasibility of conducting linkage work in this context and a basis for future research.
- It would be great for the authors to explain in more detail the selection of the 2 health facilities that were included in the study. The linkage rate of 96.3% is very high which is commendable, however would the linkage rate be similar if other health facilities were chosen instead? This would add to the study.
- A small clarification to add to the results would be the distance and whether this is measured by travel by road, therefore reflect access to services, or by overall distance.
- These next 2 suggestions are really extensions of the work that the authors could consider
   
  1. Was there any scope to record more than 1 reason for visiting the health care facility? Many of the reasons for clinic visits could simultaneously occur (ie HIV and chest infection). An ability to record more than 1 reason for a visit could greatly strengthen the work as less severe conditions could be captured (ie if someone presents for family planning reasons but also has a current skin infection, which condition would be recorded?) I’m unsure as to feasibility of recording this extra information in the study.
  2. Additionally, it would be good to have a measure if possible using the KHDSS dataset of how many young adults were registered and did not have any health facility visits. Again, I’m unsure of whether this would be possible to look at but it would help put into context the use of health care facilities in these areas.

We confirm that we have read this submission and believe that we have an appropriate level of expertise to confirm that it is of an acceptable scientific standard.

# Peer review: Reviewer response for version 1

**DOI:** 10.21956/wellcomeopenres.12195.r25438

**Published:** 2017-09-12

**Authors:** Philippe Bocquier

**Affiliations:** 1 Centre de Recherche En Démographie, Université Catholique de Louvain, Louvain‐la‐Neuve, Belgium; 2 School of Public Health, University of the Witwatersrand, Johannesburg, South Africa

This article is quite relevant to anyone who is working with HDSS data that are collected in areas served with health facilities (most HDSS are actually initiated in collaboration with these facilities). It is important for the reader to note (as mentioned by the authors) that the team build on previous experiences of HDSS-health facilities matching with children records. I would have liked the authors to reflect on a non-random selection of the two facilities chosen "on the basis of the availability of research space, youth-friendly services, a reliable power supply and a willingness to participate in the study" (p3). What would be the outcome in the other, less-welcoming health facilities? How can we evaluate this selection (probably negative) bias? This is important for generalisation of results. Another source of bias is at the individual level, when young people are identified at the reception desk. The "assessment of eligibility" may have biased the result positively. What were the eligibility criteria? What if for example a young adult declares that s/he is not living in the HDSS (or not the right age) though s/he is? I would rather not impose eligibility criteria or adopt large criteria (e.g. extending the catchment area or the age group) and then apply the algorithm on all in order to minimize the chance of false negatives. With the procedures in place, maybe only false positives can be identified, hence maybe the impressive matching success rate. The two maps in figure 2 are not easy to understand as they are no explanations attached to these maps. Also the definition of distance is not given: is it bird distance or road distance? Are people travelling by foot or by bike or car or public transport? In other words, is there a cost attached to distance? Wouldn't time to health facility be more appropriate? The analysis and discussion on reasons for visit is somewhat disconnected from the main aim of the paper. I would have preferred an analysis that shows how matching records with HDSS data can help to better understand the purpose of the visits. The paper could actually do without the analysis of reasons for visit. The paper remains interesting but precisions on the potential bias and the consequence on generalisation are needed.

I confirm that I have read this submission and believe that I have an appropriate level of expertise to confirm that it is of an acceptable scientific standard, however I have significant reservations, as outlined above.
